# An experimental model of vitreous motion induced by eye rotations

**DOI:** 10.1186/s40662-015-0020-8

**Published:** 2015-06-12

**Authors:** Andrea Bonfiglio, Alberto Lagazzo, Rodolfo Repetto, Alessandro Stocchino

**Affiliations:** Department of Civil, Chemical and Environmental Engineering, University of Genoa, Italy, Via Montallegro 1, Genoa, 16145 Italy

**Keywords:** Vitreous motion, Vitreous dynamics, Retinal detachment, Viscoelasticity

## Abstract

**Background:**

During eye rotations the vitreous humour moves with respect to the eye globe. This relative motion has been suggested to possibly have an important role in inducing degradation of the gel structure, which might lead to vitreous liquefaction and/or posterior vitreous detachment. Aim of the present work is to study the characteristics of vitreous motion induced by eye rotations.

**Methods:**

We use an experimental setup, consisting of a Perspex model of the vitreous chamber that, for simplicity, is taken to have a spherical shape. The model is filled with an artificial vitreous humour, prepared as a solution of agar powder and hyaluronic acid sodium salt in deionised water, which has viscoelastic mechanical properties similar to those of the real vitreous. The model rotates about an axis passing through the centre of the sphere and velocity measurements are taken on the equatorial plane orthogonal to the axis of rotation, using an optical technique.

**Results:**

The results show that fluid viscoelasticity has a strong influence on flow characteristics. In particular, at certain frequencies of oscillation of the eye model, fluid motion can be resonantly excited. This means that fluid velocity within the domain can be significantly larger than that of the wall.

**Conclusions:**

The frequencies for which resonant excitation occurs are within the range of possible eye rotations frequencies. Therefore, the present results suggest that resonant excitation of vitreous motion is likely to occur in practice. This, in turn, implies that eye rotations produce large stresses on the retina and within the vitreous that may contribute to the disruption of the vitreous gel structure. The present results also have implications for the choice of the ideal properties for vitreous substitute fluids.

## Background

The vitreous humour is a transparent gel mainly composed of water (about 99 %), collagen and hyaluronic acid (HA). During eye movements the adherence of the vitreous on the retina generates motion in the gel and this, in turn, produces stresses within the vitreous and on the retina. These mechanical stresses have been suggested to possibly contribute, over long time scales, to the disruption of the vitreous gel structure, leading to vitreous liquefaction and/or posterior vitreous detachment. Once the vitreous loses its homogeneity, large localised tractions can be produced on the retina [1], which are known to be often responsible for the generation of retinal tears [2]. For the above reasons, understanding the dynamics of vitreous motion secondary to eye rotations has, in the authors’ view, great conceptual and practical importance.

Vitreous mechanical properties have been measured by several investigators [3–6], in most cases employing periodic shear tests with a rheometer. Owing to its lubricating ability and its fragile and inhomogeneous network structure, obtaining reliable data of the vitreous rheology is extremely challenging. In addition, it has been shown that vitreous properties change very rapidly after dissection, further complicating *ex vivo* measurements. For the above reasons the rheological properties measured by various authors have provided very sparse data.

There have been various attempts to observe and measure vitreous motion *in vivo*. Several researchers used techniques based on ultrasound scan measurements [7–9]. In particular, Rossi et al. [9] analysed ultrasound scan films using Robust Image Velocimetry, and obtained spatial velocity fields of the vitreous humour on planes across the vitreous chamber during single ocular saccades. Another very promising technique for measuring vitreous motion *in vivo* is based on magnetic resonance imaging (MRI) and has been employed by Piccirelli et al. [10, 11] to measure vitreous dynamics during low frequency periodic eye rotations. The authors used their results to indirectly estimate vitreous rheological properties by fitting the experimental data with theoretical predictions.

Even though good progress has been made in recent years in developing measurement techniques to quantify vitreous motion, our knowledge on vitreous dynamics remains incomplete.

Various theoretical models of vitreous motion have also been proposed, which significantly contributed to improve our understanding of the phenomenon. David et al. [12] first developed a mathematical model of the vitreous motion, describing the vitreous chamber as a rigid rotating sphere and the vitreous as a linear viscoelastic material. Their work was later extended by Meskauskas et al. [13], who showed that resonant excitation of vitreous motion can possibly occur during normal eye rotation conditions. Modarreszadeh and Abouali [14] recently proposed a fully numerical model of vitreous motion accounting for a realistic geometry of the vitreous chamber.

In the past, some of the present writers have also worked on model experiments of vitreous motion e.g. [15, 16]. However, our previous experimental works were all based on the use of purely viscous fluids. The aim of the present research is to extend these studies, accounting for the effects of vitreous viscoelasticity on vitreous motion, which have been shown theoretically to play a very important role.

## Methods

### Experimental set-up

We performed laboratory experiments using an apparatus similar to that employed in previous works [15], but with some important modifications. The present model consists of a Plexiglas cylinder with an internal spherical cavity of radius *R*=1.25 cm. For the purpose of this study, we neglected the effects due to the non-sphericity of the vitreous chamber. The geometrical dimensions of the spherical cavity is approximately equal to that of the human vitreous chamber, so as to avoid any scale effects that would arise from working with a magnified eye model and would be difficult to cope with in the case of viscoelastic fluids. This is because considering for instance the case of linearly viscoelastic fluids, one should preserve between the prototype and the scaled model the values of two dimensionless parameters, e.g. the Womersley number and the ratio between the elastic and viscous components of the fluid (see for instance [17]). Once a certain fluid has been chosen for the experiments this should be accomplished by changing the model rotation frequency. However, the above two dimensionless parameters typically vary independently with the frequency, making this approach impracticable.

The eye model was mounted on a support connected to a computer-controlled motor that can induce rotations of the container with any prescribed time dependence. In this study, we only considered purely sinusoidal rotations, described by the time law

(1) $$ \varepsilon(t^{*}) = A \sin(\omega t^{*}),  $$

where *ε* is the angular displacement, *t*^∗^ is time, *A* is the amplitude of eye rotations and *ω* is their angular frequency. In the above expression, superscript asterisks indicate dimensional variables that we will scale in the following. As explained in [18] and [19], a sinusoidal law is the simplest way to represent a sequence of saccadic eye movements in both directions with a prescribed amplitude and duration. This choice is also justified by the fact that all existing data of vitreous rheological properties have been obtained considering harmonic oscillations of the vitreous. Moreover, the theoretical models by David et al. [12] and Meskauskas et al. [13], with which we will compare our results in the following, have been developed under the assumption of harmonic oscillations of the eye. Finally, this choice allows us to investigate, in the simplest possible context, the occurrence of resonance phenomena within the vitreous. We plan to extend the present work by considering the case of real saccadic eye rotations in future research.

**Fig. 1:**
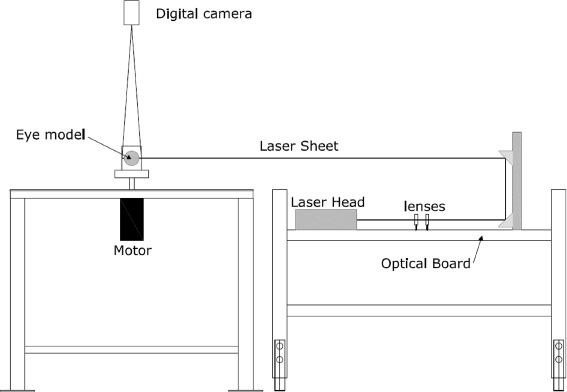
Experimental apparatus. Sketch of the apparatus, showing all the experimantal components

In each experiment, we took measurements of the instantaneous 2D velocity fields on the equatorial plane of the model, using the particle image velocimetry (PIV) technique. PIV is a fully optical method used to measure the velocity field on a plane of a moving fluid [20]. It is based on the analysis of images recorded using specifically designed digital cameras and a high-power laser light source. The fluid is seeded with small tracer particles that are assumed to be neutrally buoyant. The instantaneous velocity of the fluid is calculated analysing two frames of the moving fluid at different times and performing a cross-correlation of sub-areas of the two images. In this work, we used a XS-IR ^*TM*^ pulsed diode laser that generates a light beam with a wavelength of 795 nm (near infrared light) and a maximum pulse rate of 15 kHz. Tracer particles were hollow glass spheres with a mean diameter of 5 *μm*. The images were recorded by a X-Stream Vision ^*TM*^ XS-3 digital camera with a maximum frame rate equal to 628 Hz at a resolution of 1280×1024 pixels. The laser beam passes through an optical system that generates a laser sheet of approximately 0.5 mm thickness and a planar width sufficient to illuminate the entire equatorial plane of the eye model. A sketch of the experimental apparatus is shown in Fig. 1.

The experimental fluid employed in the present work is an artificial vitreous prepared following the instructions presented in [21]. It consists of a solution of agar powder and HA sodium salt mixed in deionised water. The resulting fluid is a viscoelastic gel, with different rheological properties depending on the concentrations of the constituents. We note that HA is a natural component of the real vitreous, which, together with the collagen, contributes to form the elastic structure of the vitreous. Agar is not present in the real vitreous; however, it represents a good proxy of the collagen fibrils in terms of elastic properties. We prepared five different solutions with different concentrations of the two components, as reported in Table 1.

**Table 1:** Main parameters of the experiments

*#* Solution	[*A* *g* *a* *r*] (*m* *g*/*m* *l*)	[*H* *A*] (*m* *g*/*m* *l*)
1	0.85	0.95
2	0.95	0.7
3	1.00	1.00
4	1.05	1.05
5	1.05	0.95

HA: hyaluronic acid

The rheological properties of the fluids were measured with an Anton Paar Physica MCR301 rheometer (torque range 10^−5^–200 mNm with resolution 1 nNm, maximum rate 3000 rpm, angular frequency 10^−5^–100 Hz), equipped with plate-cone system (diameter 50 mm, angle 2 °, gap 0.210 mm). The rheometer allowed us to perform tests in oscillatory regime at a fixed strain amplitude *γ* and variable values of angular frequency *ω*, ranging between *π*≤*ω*≤30*π* rad/s. The rheological properties of the fluid are described through the complex modulus *G*^∗^=*G*^′^+*iG*^″^ that represents the ratio between the complex amplitudes of stress and strain in an oscillatory test. *G*^′^ is a measure of the elasticity of the fluid (storage modulus) and *G*^″^ is a measure of its viscosity (loss modulus) [22].

**Fig. 2:**
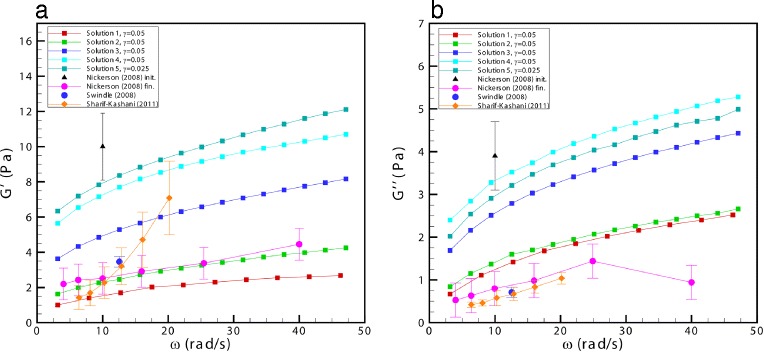
Values of the complex modulus vs frequency. Values of the moduli of the artificial vitreous (line-squares) and of the real vitreous (symbols other than squares) versus frequency, as measured *ex vivo* by various authors [4–6]: **a** *G* ^′^, **b** *G* ^″^

The values of the moduli of the fluids employed in the experiments are shown in Fig. 2, where they are compared with existing measurements of the corresponding properties of bovine and porcine vitreous [4–6]. We note that, as mentioned in the Introduction, vitreous rheological data obtained *ex vivo* are quite sparse. The fluids employed in the present work have values of the complex modulus within the range of values measured by [4–6]. The complex modulus of viscoelastic fluids is typically a function of the frequency *ω* at which the rheological test is conducted. Unfortunately, we have no way of controlling *a priori* the dependency of the complex modulus on the frequency *ω*, but we can just measure it for each particular solution. The rheological measurements show that dependence of *G*^′^ on *ω* is in qualitative agreement with the measurements by Nickerson et al. [4], but not with those by Sharif-Kashani et al. [6]. The dependency of *G*^″^ on *ω*, on the other hand, is in qualitative agreement with the existing measurements, even though our values of *G*^″^ are slightly larger than those measured *ex vivo* on the real vitreous by most authors. A notable exception, however, is the value of *G*^″^ measured by Nickerson et al. [4] just after the extraction of the vitreous form the eye (Nickerson initial values). In fact, Nickerson et al. [4] observed that the values of *G*^∗^ changed (decreased) in time after excision of the vitreous from the eye, and this leads to think that these “initial values” are likely to be the closest approximation of *in vivo* conditions. We finally note that we regard as of minor importance the fact that the artificial vitreous used in our experiments does not have the correct dependency on the frequency of oscillations. This is because each of our experiments has been performed at a fixed frequency. This is in fact an additional advantage of working with a monochromatic forcing frequency.

Particular care was taken to control and keep the fluid temperature constant during each experimental run, because the rheological properties of the fluid are highly sensitive to temperature.

In the present study, for each viscoelastic fluid, we tested the response to a periodic forcing by varying the values of the amplitude and angular frequency. The main parameters relative to each run are reported in Table 2.

**Table 2:** Solutions used in the experiments

Exp. *#*	Fluid *#*	*ε*(°)	*ω* (*H* *z*)	*T*(°*C*)
1–4	1	2–8	6.28–31.41	24
5–8	2	2–8	5.28–31.41	24
9–12	3	2–8	6.28–31.41	23
13–16	4	2–8	6.28–50.26	24
17–20	5	2–8	6.28–62.82	22

### Post-processing

The primary results from the PIV analysis of the recorded images are velocity fields $(v_{x}^{*}(x^{*}, y^{*}, t^{*}), v_{y}^{*}(x^{*}, y^{*}, t^{*}))$, where $v_{x}^{*}(x^{*}, y^{*}, t^{*})$ and $v_{y}^{*}(x^{*}, y^{*}, t^{*})$ are the velocity components in the *x*^∗^ and *y*^∗^ directions respectively at time *t*^∗^. Since we are interested in studying the flow field induced by a periodic forcing, we set the sampling rate of the PIV system in order to obtain between 20 to 50 vector fields within a single oscillation period *T*=2*π*/*ω*. The measurement of each velocity field at a specific time *t*^∗^ was repeated about 40 times and, then, an ensemble average was performed. The standard deviation of the two velocity components was found to be at most 5 % of the average velocity. The resulting vector fields were interpolated onto a polar co-ordinate system (*r*^∗^,*θ*), which is a convenient choice for the circular geometry of the domain.

In the following, we will adopt dimensionless temporal and spatial variables defined as *t*=*t*^∗^*ω*, (*x*,*y*)=(*x*^∗^,*y*^∗^)/*R* and *r*=*r*^∗^/*R*. Moreover we scale the velocity with *AωR*. The dimensionless radial (*v*_*r*_) and azimuthal (*v*_*θ*_) components of the velocity can be obtained by

(2) $$ {}v_{r}\,=\,\frac{v_{x}^{*}\cos(\theta)+v_{y}^{*}\sin(\theta)}{A \omega R}, \quad v_{\theta}\,=\,\frac{-v_{x}^{*}\sin(\theta)+v_{y}^{*}\cos(\theta)}{A \omega R}.   $$

We note that, as expected, in all experiments *v*_*r*_ is invariably much smaller than *v*_*θ*_.

We finally produced radial profiles of the circumferential velocity *V*_*θ*_(*r*,*t*) by averaging *v*_*θ*_ along the *θ*-direction, taking advantage of the axial symmetry of the flow.

For the following discussion, it is useful to introduce the integral of the dimensionless kinetic energy per unit volume of the fluid over the measurement plane, defined as

(3) $$ K= \int_{0}^{2\pi} {\int_{0}^{1}} \frac{{v_{r}^{2}}+v_{\theta}^{2}}{2} r dr d\theta.  $$

Analogous to what was done in [13], in order to obtain a synthetic parameter to quantify the intensity of vitreous motion, we introduce a new dimensionless quantity, $\overline {K}$. This is defined as the time-average of *K* divided by the analogous quantity computed for a rigid body with the same density of the fluid and undergoing the same motion, and takes the form

(4) $$ \overline{K}= \frac{8}{\pi}\int_{0}^{2\pi} K dt.   $$

Note that $\overline {K}$ is always smaller than 1 in the case of a purely viscous fluid, while $\overline {K} \rightarrow 1$ is the fluid tending to behave as a rigid body.

Our measurements show that fluid particles oscillate in time with the same frequency as that of the container and higher order harmonics, possibly generated by non-linearities, are negligible. This is not surprising since we invariably worked with small-amplitude rotations of the eye model. We can, therefore, formally write

(5) $$ V_{\theta}(r,t)=g(r)\exp(i t)+c.c.,   $$

where *c*.*c*. represents the complex conjugate. If (5) holds, we can easily calculate the shear strain generated in the fluid, which is related to *V*_*θ*_ by the following expression:

(6) $$ \gamma_{r\theta}=-\frac{iA}{2} \left(\frac{\partial{g}}{\partial{r}} -\frac{g}{r} \right) \exp(i t)+c.c.   $$

This formula is obtained by using the expression of the shear rate of strain in spherical co-ordinates and taking its integral over time.

## Results

**Fig. 3:**
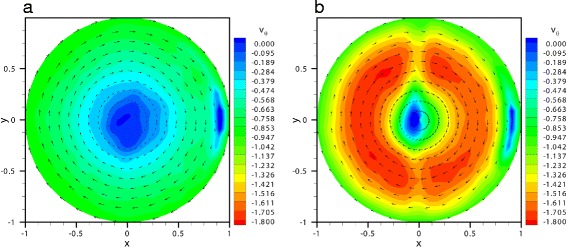
Velocity fields and contours of the magnitude of the azimuthal velocity. Velocity fields and contours of the magnitude of the azimuthal velocity component on the equatorial plane orthogonal to the axis of rotation, at *t*=*π* (i.e. the time of maximum negative angular velocity) with *A*=0.035 rad and: **a** *ω*=6.28 rad/s, **b** *ω*=43.98 rad/s. Solution *s*4. The velocity magnitude is scaled with the maximum wall velocity, the radial co-ordinate with the eye model radius and time with the inverse of the angular frequency

As mentioned in the previous section, we performed velocity measurements on the equatorial plane orthogonal to the axis of rotation where, for symmetry reasons, we assume that the out-of-plane velocity component is zero. Examples of measured velocity vector fields are given in Fig. 3a and b, for two runs performed with the same fluid at two different angular frequencies, *ω*=6.28 rad/s and *ω*=43.98 rad/s, respectively. In the figures we also plot contours of the circumferential velocity *v*_*θ*_. The two plots are relative to the time *t*=*π*, at which the angular velocity of the eye model peaks, and thus *v*_*θ*_|_*r*=1_=−1. Owing to the sphericity of the domain, contours of the circumferential velocity should be circular. The small irregularities in the contours shown in Fig. 3a and 3b are due to residual optical errors. In particular, light reflections in the region where the laser sheet enters the domain are responsible for the localised regions of low velocity that are visible on the right hand side of the plots. Moreover, some optical discontinuities are present along the diametral line corresponding to the junction of the two parts of the domain.

The two flow fields in the Fig. 3 show striking differences. The low frequency flow field, Fig. 3a, is similar to the purely viscous case, with the maximum circumferential velocity located at the boundary of the chamber, see for comparison the results discussed in [15]. On the contrary, in the high frequency case, Fig. 3a, the maximum of the circumferential velocity is no longer at the boundary, but within the core of the flow. This behaviour, which cannot occur in the case of a purely viscous fluid, is strictly related to the elastic nature of the fluid. In particular, when the container oscillates close to particular frequencies (the so-called natural frequencies of the system) resonant excitation of vitreous motion can occur. At resonance, the response of an oscillator can be much more intense than the forcing itself. In our case, this implies that particle oscillations within the core of the domain can have a larger amplitude than at the boundary.

**Fig. 4:**
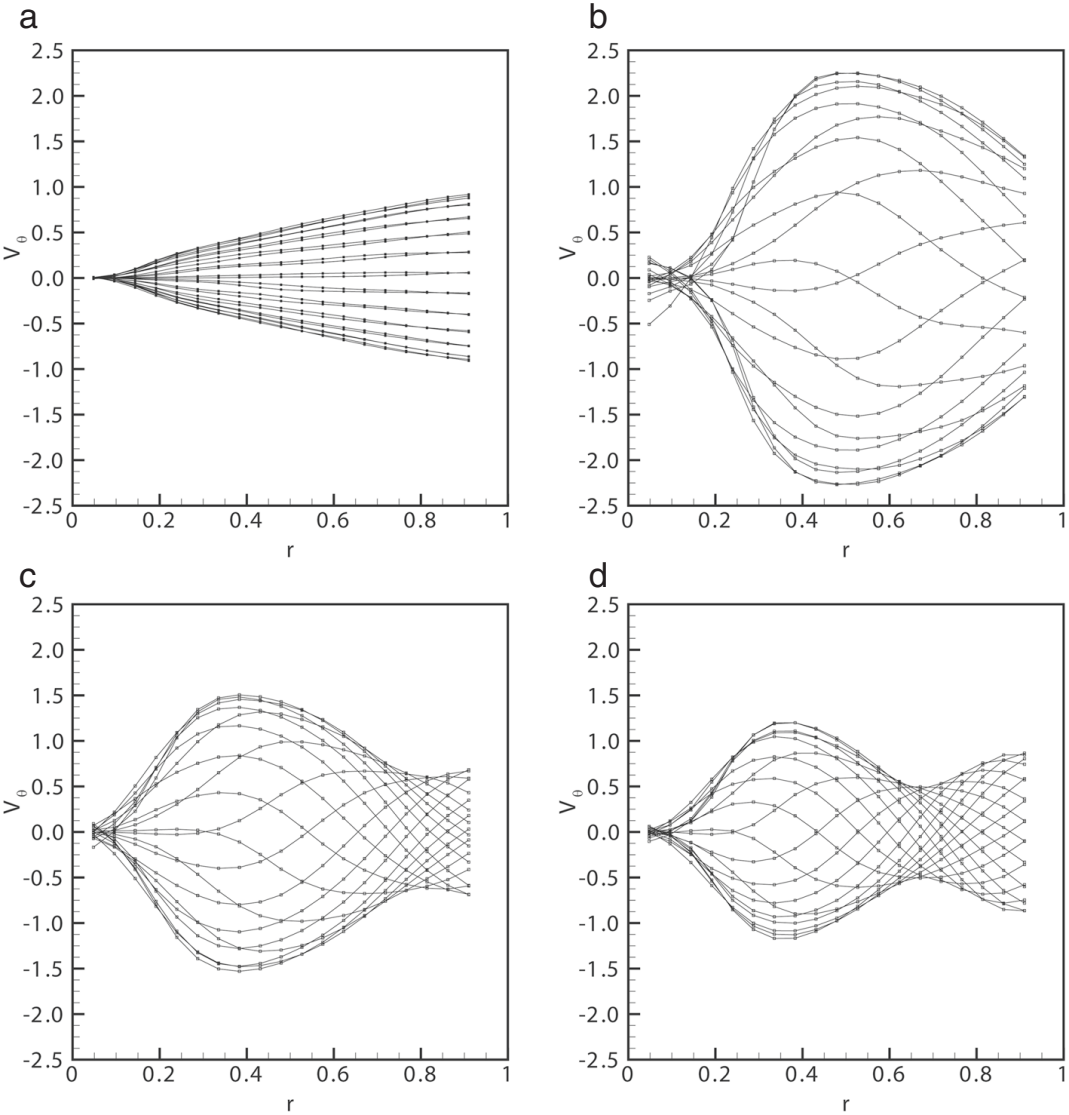
Profiles of the azimuthal velocity *V*_*θ*_. Profiles in the radial direction of the azimuthal velocity *V*_*θ*_: **a** solution *s*2, *A*=0.14 rad, *ω*=6.283 rad/s; **b** solution *s*4, *A*=0.035 rad, *ω*=50.26 rad/s; **c** solution *s*5, *A*=0.035 rad, *ω*=50.26 rad/s; **d** solution *s*5, *A*=0.035 rad, *ω*=62.83 rad/s. Each line corresponds to a different time. The velocity magnitude is scaled with the maximum wall velocity and the radial co-ordinate with the eye model radius

The possible occurrence of resonance appears even more clearly by inspection of the radial profiles of the circumferential velocity, *V*_*θ*_(*r*,*t*), some examples of which are reported in Fig. 4. Each plot refers to a different experimental run (different values of the rheological properties of the fluid and of the characteristics of motion). In the plots, each curve corresponds to a different time within the period of oscillation. The *V*_*θ*_ profiles shown in Fig. 4a, refer to a low frequency experiment, far from resonance conditions. In this case, the velocity profiles are almost straight lines. This behaviour is in agreement with theoretical predictions [12] according to which, in the limit of vanishingly small frequency (or large viscosity), the fluid would move as a rigid body. In Fig. 4b we show the velocity profiles generated close to resonant conditions. In this case the maximum fluid velocity is more than twice as large as the maximum velocity at the boundary. For frequencies above resonance, the velocity profiles assume the highly skewed shapes shown in Fig. 4c and 4d.

**Fig. 5:**
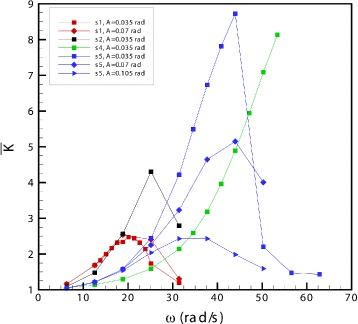
Normalised time-averaged kinetic energy $\overline {K}$ versus *ω*. Value of normalised time-averaged kinetic energy $\overline {K}$ (defined by Eq. 4) versus the angular frequency *ω*, for different values of the rotation amplitude *A* and different solutions

To quantify the intensity of vitreous motion, we made use of the normalised kinetic energy parameter $\overline {K}$, which was introduced in the Methods section. In Fig. 5, we show values of $\overline {K}$ versus the angular frequency of rotations. Each curve corresponds to a different fluid. At low frequencies, the value of $\overline {K}$ attains relatively small values. As the frequency increases and the system gets closer to resonant conditions, $\overline {K}$ also significantly grows. In all curves shown in the figure except the green one, $\overline {K}$ reaches a maximum value for a certain frequency, which is the resonant frequency of the system. As the frequency is increased above the resonant value, $\overline {K}$ decreases to relatively small values. In the case of the green curve (solution s4, as well as in other various cases not shown in the figure) all testing frequencies were smaller than the resonant value, and we were therefore not able to observe the right, decreasing branch of the curve.

The maximum value of $\overline {K}$ attained in each case depends on the rheological properties of the solution. Generally speaking, the response is more intense when the ratio *G*^″^/*G*^′^ (loss factor) is smaller. For instance, the maximum value of $\overline {K}$ is much larger in the case of solution s5 than s1, and the value of the loss factor is significantly smaller in the former case.

**Fig. 6:**
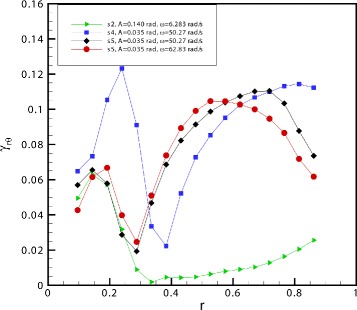
Maximum shear strain *γ*_*rθ*_ versus *r*. Maximum value of the shear strain *γ*_*rθ*_ (equation (6)) versus the radial co-ordinate *r*. The four different lines correspond to the four cases shown in Fig. 4 a- d

Finally, we present in Fig. 6 the distribution of the maximum (in time) shear strain *γ*_*rθ*_ along the radial direction, computed according to Eq. 6. The four curves reported, correspond to the experiments shown in Fig. 4a-d. In the case of the experiment at a frequency significantly lower than the resonant one (green curve) the shear strain assumes relatively small values. The peak observed at *r*≈0.2 in the green curve might be related to noise introduced after performing numerical derivatives on the measured velocity, which assumes small values in this region. On the other hand, the shear strain is much higher close to resonant conditions (blue curve) and also for frequencies higher than resonant (black and red curves), even if in the last two cases it tends to decrease close to the wall. Moreover, for these cases, a second, less intense, peak is detectable closer to the centre of the model. This local maximum appears at different times during the model oscillations with respect to the one located closer to the wall. Note that large values of the strain imply large stresses.

**Fig. 7:**
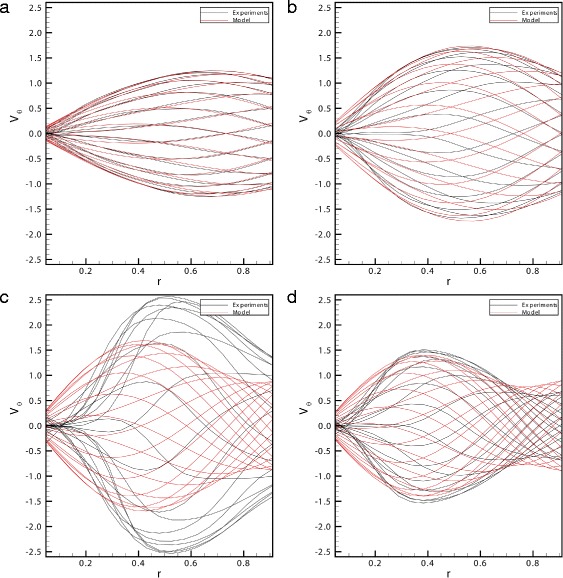
Theoretical and experimental profiles of the azimuthal velocity *V*_*θ*_. Comparison between the theoretical and experimental profiles in the radial direction of the azimuthal velocity *V*_*θ*_ for solution *s*5 and *A*=0.035 rad at different frequencies: **a** *ω*=8*π* rad/s, **b** *ω*=10*π* rad/s, **c** *ω*=14*π* rad/s, **d** *ω*=16*π* rad/s. Each line corresponds to a different time. The velocity magnitude is scaled with the maximum wall velocity and the radial co-ordinate with the eye model radius

We finally present a comparison of the theoretical predictions of Meskauskas et al. [13] with the present experimental measurements. In Fig. 7a-d we show different profiles of the azimuthal velocity *V*_*θ*_ for solution *s*5 and *A*=0.035 rad at different frequencies. In particular, the profiles of panels Fig. 7a and 7b correspond to frequency lower than the resonant frequency for that specific case, whilst the other two panels show profiles close to resonance (Fig. 7c) and for a frequency greater than the resonant one (Fig. 7d). These profiles are in very good qualitative agreement, confirming that the theoretical model of Meskauskas et al. [13] well describes the behaviour of the viscoelastic fluid under oscillating forcing. However, the more significant differences are detectable close to resonance. These discrepancies may be attributed to some aspects that are not included in the theoretical model, such as spatial and temporal variability of the fluid properties. In fact, the rheological tests showed that the complex modulus of the fluid depends on the rate of shear. The latter changes in space and time and the complex modulus does so accordingly. This effect is not accounted for in theory and may play a more important role than nonlinearity as discussed by Repetto et al. [17], who showed that nonlinear corrections to the azimuthal velocity profiles would appear at the third order (in the small strain) and it is thus likely to contribute little to the measured velocity profiles.

## Discussion and conclusions

In the present work, we presented an experimental model of the vitreous humour motion induced by eye rotations. Understanding the dynamics of vitreous humour is clinically relevant since indications exist that the possible occurrence of retinal tears and, eventually, retinal detachment are related to mechanical actions exerted by the vitreous on the retina. Moreover, various authors e.g. [23, 24] hypothesised that mechanical stresses within the vitreous might contribute to the disruption of the gel structure, over long times scales. This could explain the occurrence of liquefaction that is typically observed with advancing age.

We modelled the vitreous chamber as a rigid sphere and we filled it with an artificial vitreous with viscoelastic properties. In particular, we chose a fluid with rheological properties similar to those of the real vitreous, measured *ex vivo* by various authors. We remark, however, that there is still a lot of uncertainty concerning the mechanical properties of the vitreous, and the available measurements provide values within a very wide range. In order to keep the problem as simple as possible, yet retaining some important ingredients, we modelled eye movements as sinusoidal periodic rotations and investigated the role of frequency on the results.

Various experimental models of vitreous motion induced by eye rotations have been proposed in the past, however, to our knowledge, this is the first case in which a viscoelastic fluid has been employed. Our results show that this is a crucial ingredient. In fact, for all solutions employed to create the artificial vitreous, resonance excitation of vitreous motion was observed for values of the frequency that are typical of eye rotations. With resonance we refer to tendency of the fluid to oscillate with larger amplitudes at some frequencies than at others. Such frequencies are named “resonant frequencies”. At resonance, the system stores and easily transfers energy between two different storage modes. In the case of the motion of a fluid completely filling a closed rotating domain, which is considered in the present work, viscoelasticity is a necessary ingredient to possibly generate resonance phenomena. This is because the system needs to be able to store elastic energy during certain phases of motion, which is then transformed into kinetic energy at other times. We note that in this work we have not described the transient conditions during which the oscillation amplitude progressively grows, starting from rest, until periodic flow conditions are reached. If the eye model rotates at a frequency close to resonant, oscillations in the core of the domain can have a significantly higher amplitude than at the wall. This is a relevant phenomenon to investigate because, if resonance occurs, particularly large values of the stress are attained on the retina and within the vitreous. The order of magnitude of natural frequencies investigated in the experiments are comparable with typical frequencies associated with saccadic eye movements, as derived following the same approach as in [18, 19], where the angular frequency for sinusoidal oscillations was computed as *ω*=*π*/*D*, where *D* is the saccade duration.

Resonant excitation of vitreous motion *in-vivo* has not been observed, and we are not yet in the position of assessing whether it can actually occur. This is due to a lack of *in-vivo* data on vitreous dynamics. As mentioned in the Introduction, Piccirelli et al. [11] measured vitreous motion using an MRI based technique. In their experiments, patients were asked to rotate their eyes following a target performing a sinusoidal motion. However, they only considered low frequencies, at which our experimental model does not predict the occurrence of resonance. Rossi et al. [9] used an ultrasound technique to measure vitreous dynamics. Since in their investigation they asked patients to perform single saccadic rotations, it is difficult to assess from their data whether natural frequencies of the system exist that could be resonantly excited by periodic rotations. Moreover, in order for the vitreous to be visible at the ultrasound scan, the authors had to restrict their study to patients with a somewhat degenerated vitreous structure. This lack of information points to the need of new *in vivo* measurements of vitreous motion. If resonance can actually occur during normal eye movements, it would imply that the vitreous and the retina can be subjected to much higher mechanical stimuli than we would expect.

The present results have practical value also from another point of view. A lot of effort has been devoted in recent years to the identification of the ideal material to be employed as a vitreous substitute. Various authors regard fluid elasticity as being very important to avoid excessive flow within the vitreous chamber [25–27]. This is certainly true; however, if the ratio between fluid viscosity and elasticity is not large enough, resonance can occur and produce undesirably large values of the stress in the fluid and on the retina. This should be kept in mind in the design of the ideal artificial vitreous.
